# The UCSC Genome Browser database: 2022 update

**DOI:** 10.1093/nar/gkab959

**Published:** 2021-10-28

**Authors:** Brian T Lee, Galt P Barber, Anna Benet-Pagès, Jonathan Casper, Hiram Clawson, Mark Diekhans, Clay Fischer, Jairo Navarro Gonzalez, Angie S Hinrichs, Christopher M Lee, Pranav Muthuraman, Luis R Nassar, Beagan Nguy, Tiana Pereira, Gerardo Perez, Brian J Raney, Kate R Rosenbloom, Daniel Schmelter, Matthew L Speir, Brittney D Wick, Ann S Zweig, David Haussler, Robert M Kuhn, Maximilian Haeussler, W James Kent

**Affiliations:** Genomics Institute, University of California Santa Cruz, Santa Cruz, CA 95064, USA; Genomics Institute, University of California Santa Cruz, Santa Cruz, CA 95064, USA; Genomics Institute, University of California Santa Cruz, Santa Cruz, CA 95064, USA; Medical Genetics Center (Medizinisch Genetisches Zentrum), Munich 80335, Germany; Genomics Institute, University of California Santa Cruz, Santa Cruz, CA 95064, USA; Genomics Institute, University of California Santa Cruz, Santa Cruz, CA 95064, USA; Genomics Institute, University of California Santa Cruz, Santa Cruz, CA 95064, USA; Genomics Institute, University of California Santa Cruz, Santa Cruz, CA 95064, USA; Genomics Institute, University of California Santa Cruz, Santa Cruz, CA 95064, USA; Genomics Institute, University of California Santa Cruz, Santa Cruz, CA 95064, USA; Genomics Institute, University of California Santa Cruz, Santa Cruz, CA 95064, USA; Genomics Institute, University of California Santa Cruz, Santa Cruz, CA 95064, USA; Genomics Institute, University of California Santa Cruz, Santa Cruz, CA 95064, USA; Genomics Institute, University of California Santa Cruz, Santa Cruz, CA 95064, USA; Genomics Institute, University of California Santa Cruz, Santa Cruz, CA 95064, USA; Genomics Institute, University of California Santa Cruz, Santa Cruz, CA 95064, USA; Genomics Institute, University of California Santa Cruz, Santa Cruz, CA 95064, USA; Genomics Institute, University of California Santa Cruz, Santa Cruz, CA 95064, USA; Genomics Institute, University of California Santa Cruz, Santa Cruz, CA 95064, USA; Genomics Institute, University of California Santa Cruz, Santa Cruz, CA 95064, USA; Genomics Institute, University of California Santa Cruz, Santa Cruz, CA 95064, USA; Genomics Institute, University of California Santa Cruz, Santa Cruz, CA 95064, USA; Genomics Institute, University of California Santa Cruz, Santa Cruz, CA 95064, USA; Genomics Institute, University of California Santa Cruz, Santa Cruz, CA 95064, USA; Genomics Institute, University of California Santa Cruz, Santa Cruz, CA 95064, USA; Genomics Institute, University of California Santa Cruz, Santa Cruz, CA 95064, USA

## Abstract

The UCSC Genome Browser, https://genome.ucsc.edu, is a graphical viewer for exploring genome annotations. The website provides integrated tools for visualizing, comparing, analyzing, and sharing both publicly available and user-generated genomic datasets. Data highlights this year include a collection of easily accessible public hub assemblies on new organisms, now featuring BLAT alignment and PCR capabilities, and new and updated clinical tracks (gnomAD, DECIPHER, CADD, REVEL). We introduced a new Track Sets feature and enhanced variant displays to aid in the interpretation of clinical data. We also added a tool to rapidly place new SARS-CoV-2 genomes in a global phylogenetic tree enabling researchers to view the context of emerging mutations in our SARS-CoV-2 Genome Browser. Other new software focuses on usability features, including more informative mouseover displays and new fonts.

## INTRODUCTION

The UCSC Genome Browser provides a tool to examine and explore biological data in relation to the human genome and the genomes of many other organisms. The site's vast data collection, referred to as annotations or tracks, are available on the human genome, while we also provide a means to display data for any genome assembly. The most notable improvements from the past year include a more informative mouseover display, a new representation of variants, and a new Track Sets feature to support clinical data interpretation. We have also expanded our site's popular BLAT and PCR tools to a new collection of Genome Archive (GenArk) assembly hubs.

## BACKGROUND

The UCSC Genome Browser's variety of tools aid in the interpretation of genomic data. The primary tool used by many researchers is a base-by-base visualization of DNA sequence, where additional PCR and BLAT tools aid in preparing primers for experiments or looking for DNA motifs. One of the Browser's most valuable services is enabling the discovery of connections with annotations generated by other researchers, where laboratory experiments from around the world are uploaded and aligned to specified coordinate ranges. The site's tools are engineered to allow users to attach data generated in their own lab through mechanisms known as custom tracks, track hubs, and assembly track hubs, enabling visualization and tool operations on files hosted online. For example, with tracks showing the alignment of other organisms to the human genome, such as mouse or zebrafish, visitors to the Browser can see their data in the context of evolution right on their screen.

Most data are displayed graphically in the Browser as horizontal ‘tracks’ over the genome sequence representing annotations aligned to the coordinate space. The original annotation blocks are known as BED (Browser Extensible Data) or bigBed tracks. The ‘big’ prefix in bigBed and other ‘big’ files refers to remotely-hosted binary-indexed genomic data (1). Many veteran users of text-based custom tracks have experienced challenges converting their data into these binary versions, so we recently released a new blog post, https://bit.ly/UCSC_blog_bigBed, to help guide labs through these steps. Many users are also not aware of advanced ways to share their data, so another recent blog post, https://bit.ly/UCSC_blog_sharing, illustrates examples of using URL parameters to attach custom tracks, or even to attach track hubs on top of assembly hubs, with a single link.

Data can be directly downloaded from a dedicated server, https://hgdownload.soe.ucsc.edu. Almost all data, including binary-indexed versions attached via remotely hosted hubs, can be programmatically extracted through an API at https://api.genome.ucsc.edu (2), or accessed within a user interface on our Table Browser, https://genome.ucsc.edu/cgi-bin/hgTables (3).

## NEW GENOME BROWSER DATA

In the past year, much of our newly released data focused on supporting new assemblies, clinical variant interpretation, and the SARS-CoV-2 browser, each discussed in separate sections below. A complete list of new and updated track data is available in the Supplementary Table S1. Not listed in the supplemental section are the many new liftOver files generated to map the differences between assemblies, whether one human assembly to the next, or between different species. For a new mouse GRCm39 (mm39) assembly, we released 35 of these liftOver files from mouse to various other species, such as zebrafish, rat, and human. For the human hg19 and hg38 assemblies, we released a composite of two alignment tracks created using NCBI’s ReMap tool alongside a UCSC LiftOver track that enables mapping comparisons. We also produced many of these liftOver files between specific species as requested by users on our mailing list, with all files available on our download server.

### New assemblies

To provide some context for our new GenArk assembly hubs, the UCSC Genome Browser makes a distinction between internal and external assemblies. Internal assemblies are integrated into our tools by our engineering team and are supported by a combination of local MySQL databases and indexed binary data files. Originally, all assemblies available in the Browser were internal. As sequencing and assembly technology became more widespread, however, it became more important to support the visualization of new assemblies without our staff involvement. We introduced the capability for externally hosted assemblies which are provided entirely over the Internet and are managed by external data producers. These external assembly track hubs do not depend on our internal MySQL databases as all of the data are provided through a linked set of online text and binary files (1,4,5). The new GenArk hubs exist external to the main UCSC site, hosted on our separate dedicated download server.

#### New internal assemblies and data

This year we released a handful of new internal assemblies, notably mouse GRCm39 (mm39), Gorilla (gorGor6), Bonobo (panPan3), Marmoset (calJac4), Dog (canFam4 and canFam5), Rat (rn7), and Hawaiian monk seal (neoSch1).

#### New external assemblies and data

**Figure 1.**
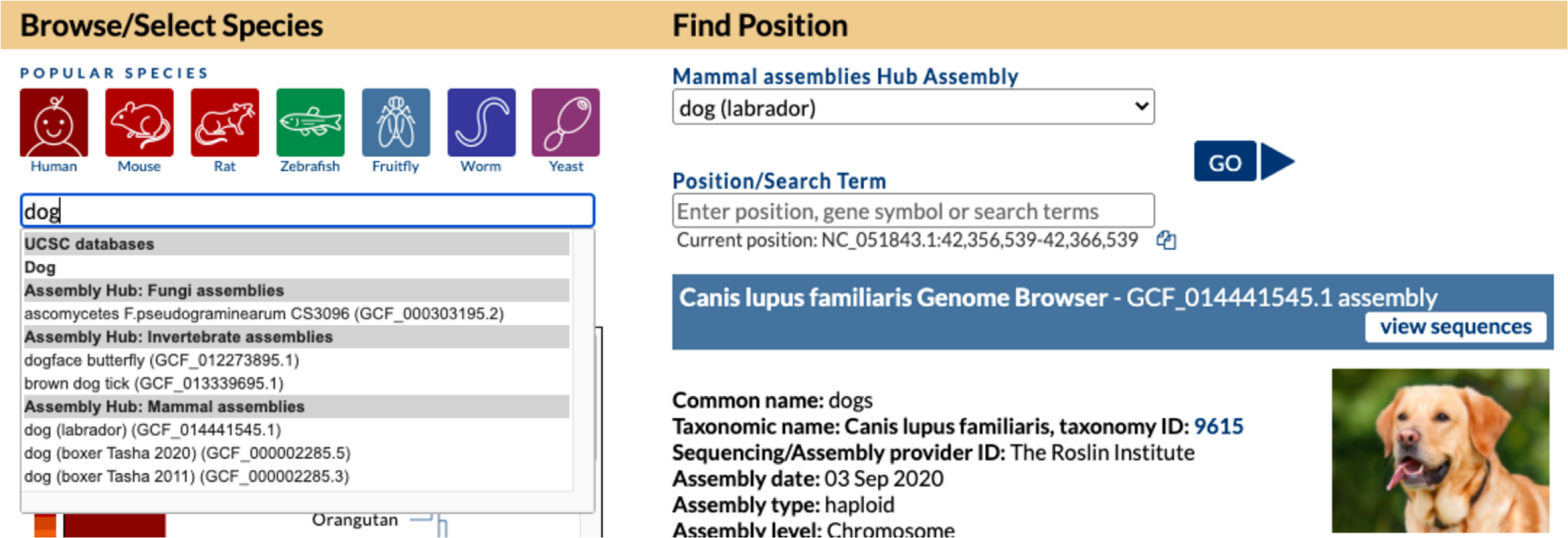
On the Genome Browser Gateway page, a variety of genome assemblies are available. Organisms can be located using a phylogenetic tree below the search box on the left side of the page or via text search. The gateway search box also includes results for public assembly hubs, including from the new GenArk Genome Archive. Here, a search for ‘dog’ returns an internal *Canis lupus familiaris* result as well as results for several specific dog breeds in external public assembly hubs with labrador (GCF_014441545.1) selected.

We released a collection of >1300 non-human Genome Archive public assembly hubs. The GenArk genomes are sourced from NCBI RefSeq, the Vertebrate Genomes Project (VGP) (6) and other projects. These new assemblies are discoverable by searching on the ‘Public Hubs’ page under the ‘My Data’ menu, or on our ‘Genomes’ gateway page (Figure 1). All GenArk assemblies come ready-for-use with several pre-computed annotation tracks and new this year is the ability to align genomic sequence to the assembly using our BLAT alignment and In-Silico PCR tools. The resource can be easily expanded in the future, where an automated pipeline can generate similar files for new assemblies as users request assembly browsers for other GCF-accessioned genome assemblies. Individual GenArk assemblies can also be launched directly with short links such as https://genome.ucsc.edu/h/GCF_014441545.1 where the GCF-value refers to the NCBI accession for that assembly, in this case, a labrador dog genome.

### New and updated clinical data

To better support personal genomics data and clinical geneticists, we added Whole Exome Sequencing (WES) probesets tracks for hg38 and hg19. The new Exome Sequencing Probesets collection includes data for exon-capture kits from Illumina, Agilent, Roche, IDT, Twist, and MGI (BGI). These 78 new subtracks assist with the interpretation of sequencing results. For example, missing data from an exon may represent a deletion in a clinical sample, indicating a pathogenic state, or could be due to the failure of a particular probeset to capture the exon from a specific gene isoform.

To visualize phased personal genomics data, this year we released two new track sets featuring family trios from the Genome in a Bottle Consortium (7) and 1000 Genomes Project (8). These tracks use a track type developed last year (vcfPhasedTrio) (9), which display child variants flanked by variants from both parents, enabling distinguishing between inherited variants and those arising de novo in the child. The tracks come with new abilities to drag and reorder the arrangement of the trios and to color the functional effect of mutations.

Other new clinical tracks include the dbVar (10) Common Structural Variants track that aggregates data from many sources. Also a new hg19 gnomAD pext (proportion expression across transcript scores) (11) track aids in investigating alternative splicing and the clinical assessment of rare variants (12). Another new clinical track worth highlighting is the Combined Annotation Dependent Depletion (CADD) track (13). The CADD track supplies a deleteriousness score of single nucleotide variants, where CADD scores correlate with the pathogenicity of both coding and non-coding variants and experimentally measured regulatory effects (14). The CADD track features six signal subtracks, four for every possible mutation (A, C, G, T) and two more for insertions and deletions. Similar to the CADD track, a new REVEL (rare exome variant ensemble learner) track predicts the pathogenicity of missense variants for every possible basepair change across all coding sequences (15).

Clinical data improvements were not limited to new tracks; we have also updated a number of existing clinical tracks with new features. As an example, our ClinVar SNVs and ClinGen tracks now include a more detailed mouseover display to facilitate the faster assessment of phenotype and clinical significance. An optional new feature also collapses lengthy Copy Number Variants (CNVs) that span a genomic region larger than the current window. For these tracks, we colored CNVs in a gradient according to clinical importance and added an extensive set of filtering options, including by clinical significance (benign, conflicting, etc.), by allele origin (somatic, germline, *de novo*, etc.), and by molecular consequence (stop-loss, nonsense, intron variant, etc.). These mouseover and filter enhancements were added to several other clinical tracks as well.

### SARS-CoV-2 data

One month before the 2020 pandemic was declared, we built a SARS-CoV-2 assembly browser (9,16) to assist the scientific community with education and research. Information about SARS-CoV-2 has rapidly evolved over the past year, and we released new annotations as data became available. A ‘UCSC COVID-19 Research’ link under the ‘Projects’ menu on our home page provides access to news summaries for released tracks, https://genome.ucsc.edu/covid19.html#news, and new SARS-CoV-2 tracks are flagged in the Supplementary Table S1. For the benefit of new users, we added a database-specific introduction, https://genome.ucsc.edu/goldenPath/help/covidBrowserIntro.html, that provides an overview of our SARS-CoV-2 resources and a video, https://bit.ly/ucscVid20, introducing the Browser to virologists.

### New public hubs

Public hubs allow external groups to package data into a collection of online files and make their findings discoverable on our public hubs page. Researchers can contact the Genome Browser team and request that we add their hub once they have fully documented it. New public hubs added this past year are listed in Table 1.

**Table 1.** New public hubs, 2020–2021

Track Hub Name	Provider	Assemblies
ALFA (Allele Frequency Aggregator)	NCBI, dbGaP	hg19, hg38
Digital genomic footprinting	Altius Institute (Jeff Vierstra)	hg38
ENCODE DNA, RNA, and Integrative cCRE Hubs	UMass Medical School (Zhiping Weng)	hg19, hg38, mm10
Exp/Meth VNTR	Icahn School of Medicine at Mount Sinai (Andrew Sharp)	hg19, hg38
GENCODE Updates	GENCODE	hg38, mm10, mm39
Genome Archive (GenArk)	NCBI/VGP	1,331 assemblies
PhyloCSF++	Johns Hopkins University (Steven Salzberg and Christopher Pockrandt) and the Seoul National University (Martin Steinegger)	rn6, fr3, gasAcu1, tarSyr2, sacCer3
T-cell ChIP and ATAC seq	Scripps Research (Matthew Pipkin)	mm10
UniBind 2021	University of Oslo (Rafael Riudavets Puig)	hg38, mm10, ce11, dm6, danRer11, sacCer3, rn6, araTha1

## NEW GENOME BROWSER SOFTWARE

This past year we created a new Recommended Track Sets feature that facilitates the interpretation of variants in the clinic. We also enhanced the lollipop display to aid the understanding of variant data. In support of SARS-CoV-2 research, we integrated a tool that enables scientists to visually interpret new virus variants. Other software enhancements include an improved user interface with more informative mouseovers and new optional fonts. New advanced settings for track hubs allow for filtering items, accessing extra data fields, and adding PCR searches to assembly hubs.

### Recommended track sets

**Figure 2.**
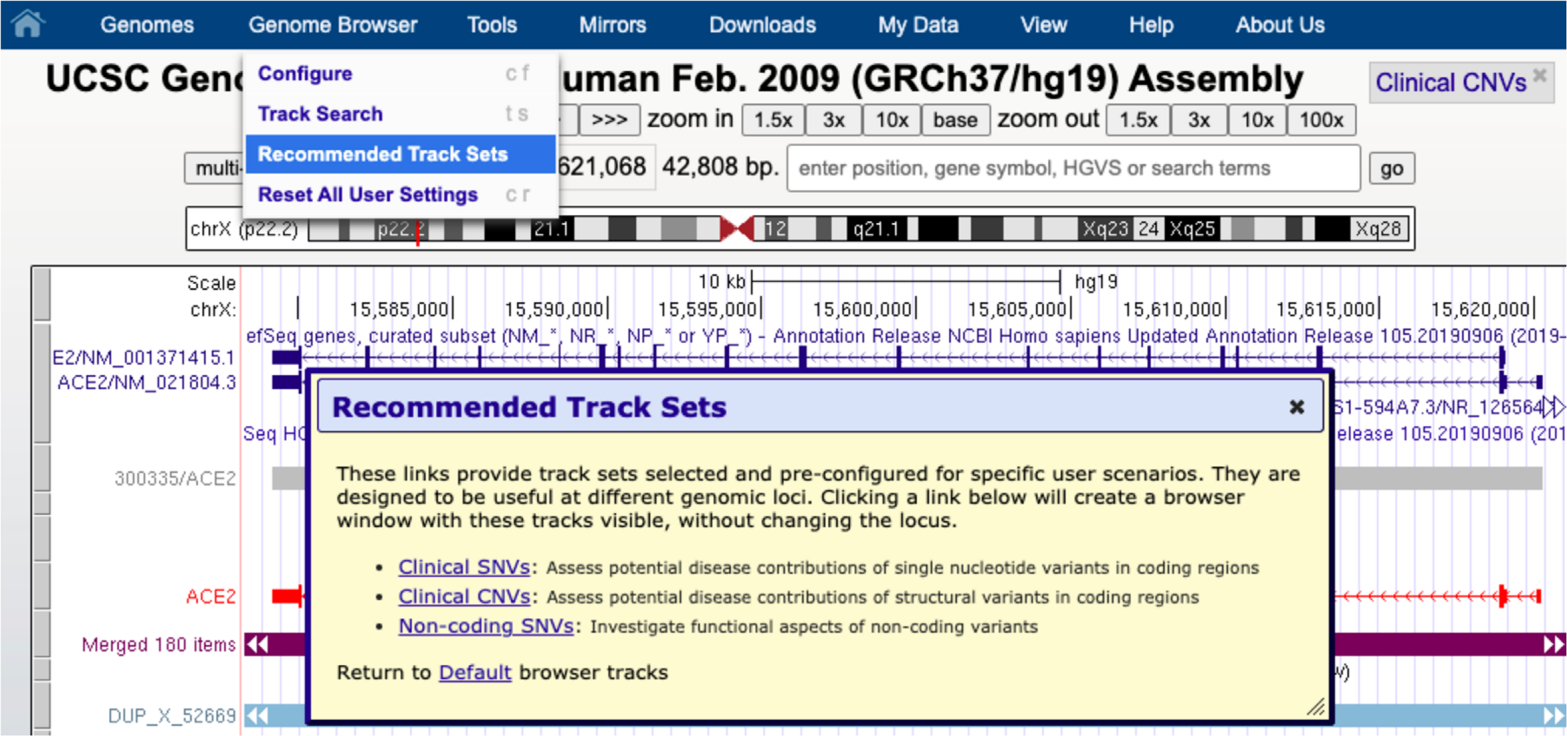
New recommended track sets feature on the human hg19 assembly. Selection in the top blue menubar under ‘Genome Browser’ launches a dialog box offering three pre-configured track sets. These collections support clinical scenarios for interpreting variants, where a user can swap from one view to another view without changing position.

A new Recommended Tracks Set feature, available on GRCh37/hg19, collects related clinical tracks for specific use cases. Track Sets swap out the current annotations a user may be viewing at the current genomic position for a recommended set, with the first versions relevant to different clinical scenarios (17). These track sets (Figure 2) focus on data relevant to investigating single nucleotide variants in coding regions (clinical SNVs), structural copy number variants (clinical CNVs), and functional aspects of non-coding variants (non-coding SNVs).

### Updated variant display

**Figure 3.**
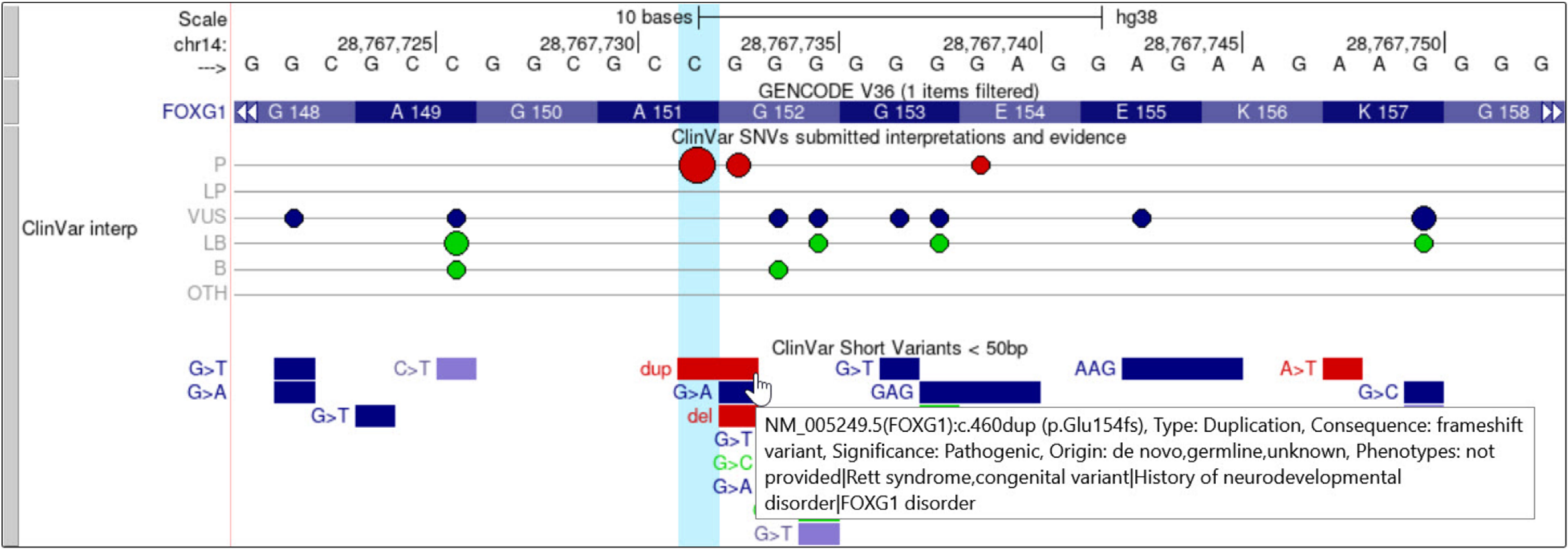
The human hg38 ClinVar Interpretations and related ClinVar Short Variants track showing part of the FOXG1 gene. ClinVar Interpretations is the first track to use our bead graph display, which is a variation of our existing lollipop display. The size of the bead on the line represents the number of submissions at that genomic position. The color of the beads further aids to distinguish the categories (red = pathogenic or likely pathogenic, blue = variant of unknown significance, green = benign or likely benign). The image also demonstrates another improved feature in the ClinVar Short Variants track: expanded support for complex mouseover text on individual items, also available for track hubs.

In previous years we added a lollipop (bigLolly) (2,9) display for variants where height and color help emphasize small, high-scoring variants in regions with thousands of annotations. This year we improved several aspects of the lollipop display to help convey important information at a glance. Individual lollipops now have a radius that scales according to a metric for the associated variant, such as the ratio of total studies providing supporting evidence. A new ‘beads on a string’ display option can be seen in the new ClinVar (18) Interpretations track (Figure 3). The size of each bead represents the number of submissions for that variant, and variants are grouped into horizontal lines according to their ClinVar classification (pathogenic, likely pathogenic, uncertain, etc.). More information on creating and using lollipop tracks can be found at a new help page: https://genome.ucsc.edu/goldenPath/help/bigLolly.html.

### SARS-CoV-2 phylogenetics

**Figure 4.**
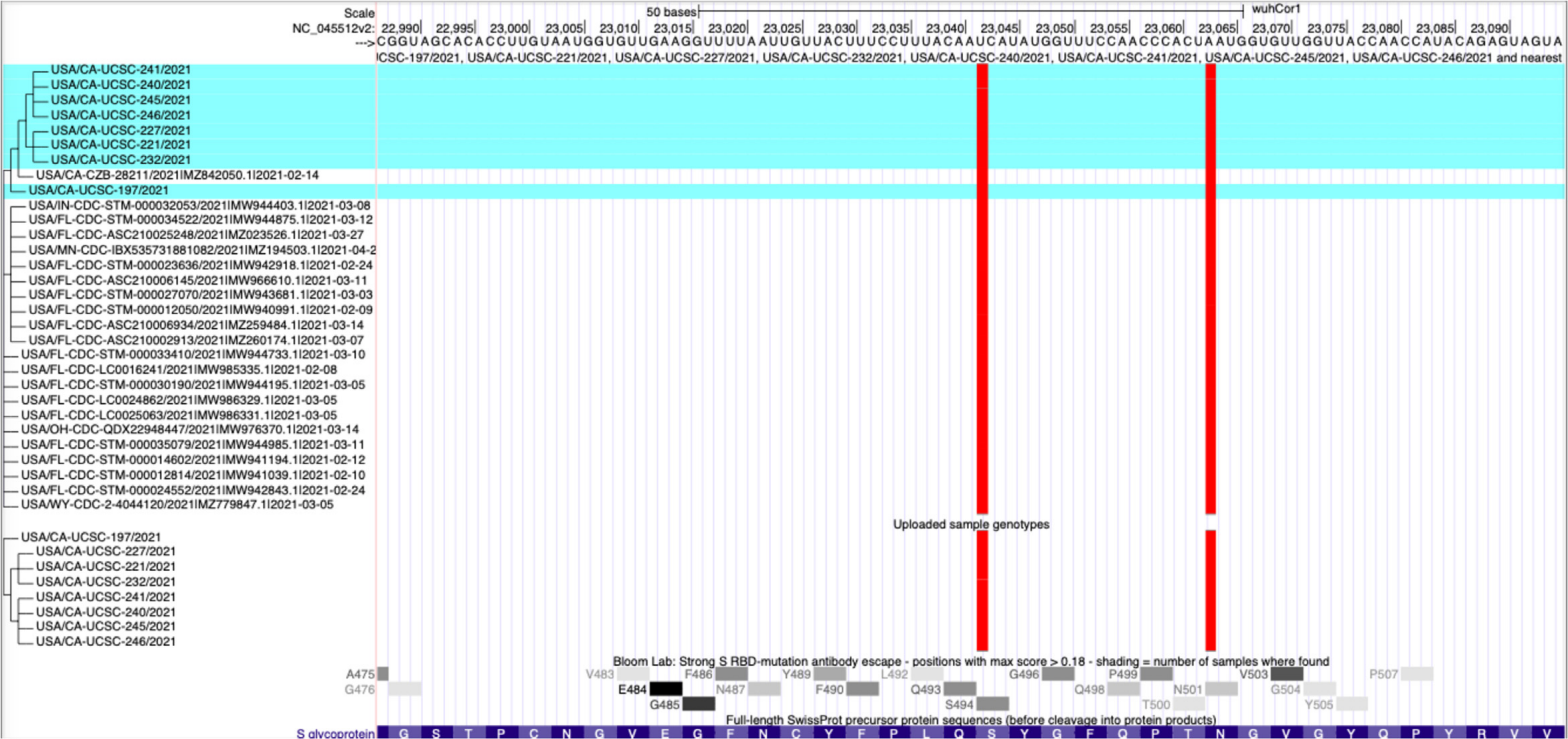
Sequences uploaded to UShER are highlighted in a blue horizontal background alongside their most closely related public sequences, at the top of the tree in this image. This allows researchers to investigate relationships among samples, and evaluate their mutations using information from other tracks such as the antibody escape track, second from the bottom. The figure displays two spike protein mutations that overlap with items in the antibody escape track where mutations are colored red for non-synonymous changes.

To help researchers quickly grasp the potential impact of new virus variants we released a new web interface to the Ultrafast Sample placement on Existing tRee (UShER) tool (19). UShER places novel SARS-CoV-2 genome sequences onto an existing SARS-CoV-2 phylogenetic tree and extracts subtrees showing the new genome sequences alongside their closest known relatives (Figure 4). The web interface generates custom tracks for the uploaded variants and subtrees, downloadable summary files, and JSON files for display by nextstrain.org. A training module, ‘Real-time phylogenetics with UShER’, is available at the Centers for Disease Control website, https://www.cdc.gov/amd/training/covid-19-gen-epi-toolkit.html, and helps guide scientists on how UShER can speed the analysis of new variants.

### Enhanced signal display

**Figure 5.**
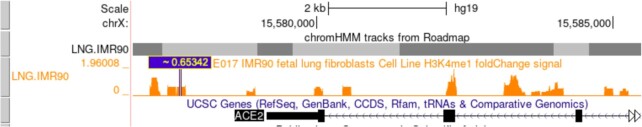
The mouseover display in a blue box (∼0.65342) gives instant access to data values contained in a signal track. The tilde indicates that multiple values are combined at the location due to data compression at this visibility level. Here the value indicates H3K4me1 scores from human fetal lung fibroblasts, from data in the Roadmap Epigenomics Integrative Analysis public hub, one of the most accessed public hubs. This feature is now available on all signal tracks.

All signal tracks now have a new mouseover pop-up that shows the score in the current position as the mouse moves over the data display. The feature gives the score value corresponding to the cursor location for the signal track (Figure 5). The utility of this feature is noteworthy in our new variant pathogenicity CADD score track (13), as it enables users to easily obtain the exact numerical value at a nucleotide, a documentation requirement for clinical genetics curators.

### New font options

**Figure 6.**
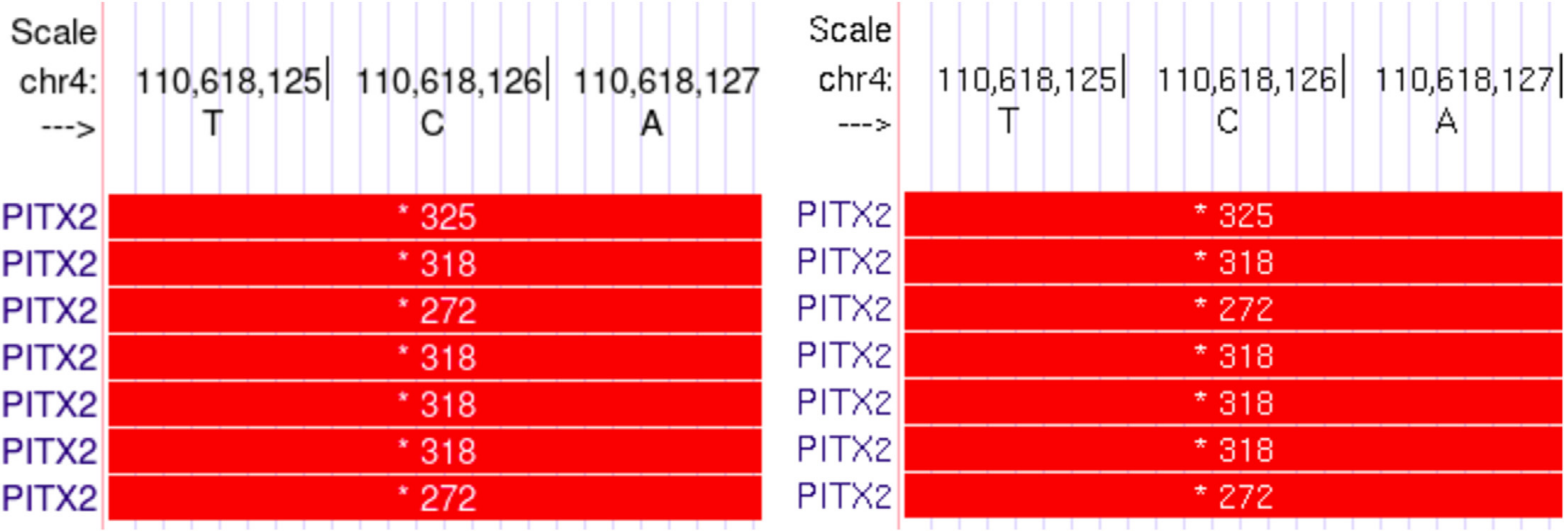
Comparison of the display quality between the new fonts (vector-based with anti-aliasing) on the left showing a stop codon for the PITX2 gene and the old (bitmap-based) fonts on the right showing the same location.

For the first time, options for different vector-based and anti-aliased fonts are available for the main Browser display (Figure 6). Today's screens allow bigger font sizes and anti-aliasing makes these more readable. Five fonts from Avant Garde to Zapf Chancery with style options such as bold and italic are available. To find the font selection options use the top blue menu bar to access the Configure page under the ‘Genome Browser’ selection.

### Updated multi-region access

**Figure 7.**
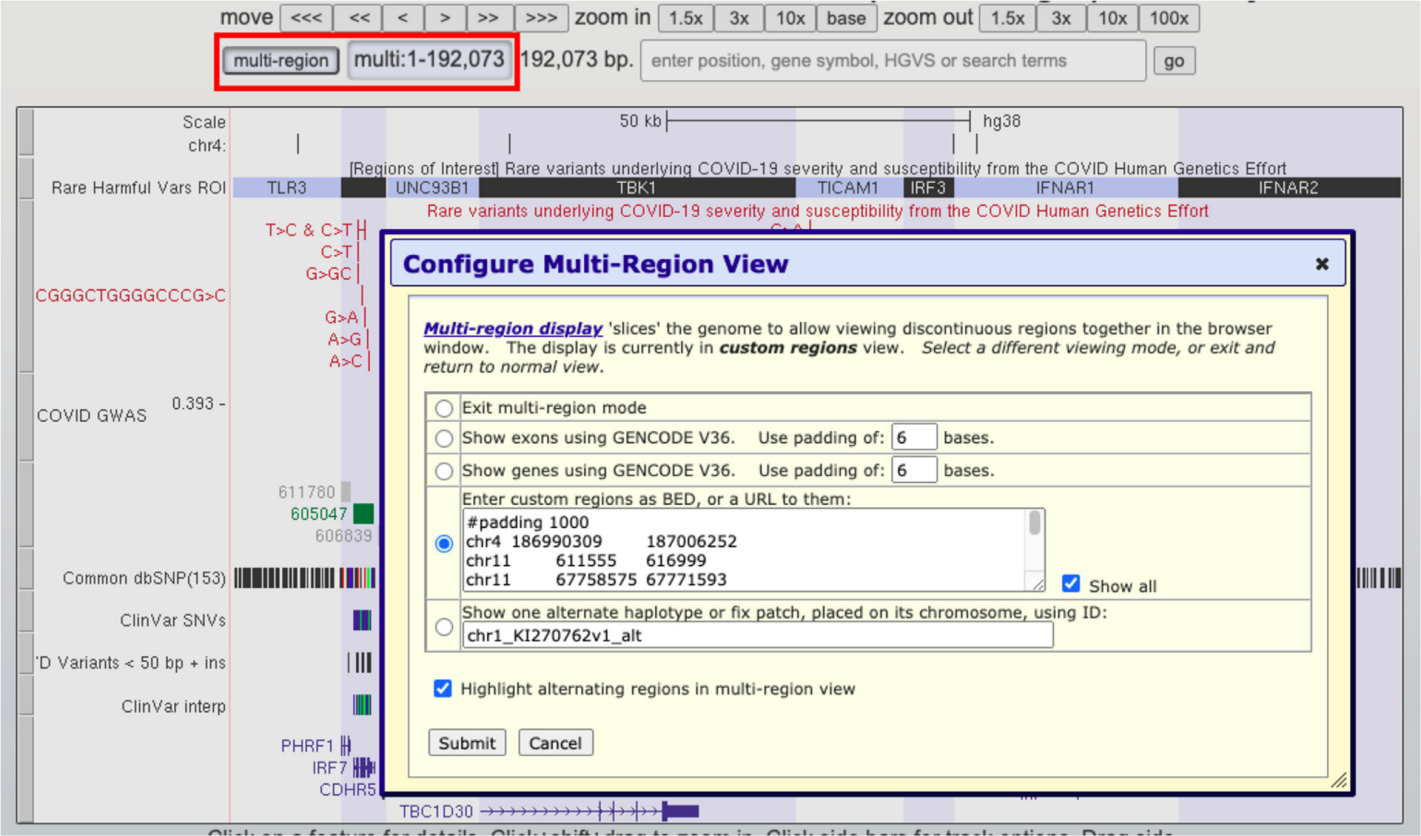
The new location of the multi-region button adjacent to the position text box (red rectangle). The button is highlighted when multi-region is activated, and the configure screen now includes a prominent help link at the top. The image in the background shows data from the rare harmful variants track associated with severe COVID outcomes, displaying eight genes across five different chromosomes, highlighted in alternating white or blue backgrounds.

The multi-region button has been relocated next to the position text box to facilitate faster toggling between states and to improve discovery by users. Multi-region allows users to vertically slice their tracks into a variety of different modes, including ‘exon-only,’ so only the portions of track annotations that fall within specified regions are shown. When the feature is activated, the button is prominently highlighted to alert users. This update to the multi-region interface was motivated in part to aid users in the display of a new Rare Harmful Variants track, which shows 23 rare variants associated with severe COVID outcomes from the COVID Human Genetic Effort (20). The track employs a new feature to enter multi-region mode, where a single click will show sections of five chromosomes at once to see all of the variants, which are scattered across eight human genes (Figure 7).

### Enhanced track hub filters

We implemented new types of filtering on additional fields of numerical and text annotations in bigBed files. These filters allow users to zero in on specific elements of interest, which can often be lost in a larger ocean of data. A new quick start guide, https://genome.ucsc.edu/goldenPath/help/hubQuickStartFilter.html, provides comprehensive illustrations of how hub developers can take advantage of these new filters.

### Track hub settings for conveying more information

Two settings have been added to give hub creators better control over the display of their data with complex additional fields. The first is a new mouseover setting, https://genome.ucsc.edu/goldenPath/help/trackDb/trackDbHub.html#mouseOver, to control the pop-up text shown when moving the mouse cursor over items in a track. The new setting can draw from multiple fields of the track data simultaneously. An example of this is seen in the ClinVar Short Variants track (Figure 3).

The second new setting (extraTableFields) allows the details pages for individual bigBed track items to display text accessed from additional files. The new option requires a URL or relative path to a table or file, allowing for much more information to be presented when the user clicks into the details page of a specific item. An example of this feature can be seen in the gnomAD Variants Track (11). By clicking into an item, two tables titled ‘Variant Effect Predictor’ and ‘Population Frequencies’ display complex data that are not contained within the original track file, but are instead sourced via the new extraTableFields setting, https://genome.ucsc.edu/goldenPath/help/trackDb/trackDbHub.html#extraTableFields.

### Dynamic PCR and BLAT for assembly hubs

New assembly hub features were added in the process of developing the GenArk assembly hubs, including in-silico PCR, which can be invoked with the setting ‘isPcr’ when a BLAT server is running. In connection with that setting, we developed a new dynamic PCR and BLAT feature to provide sequence alignment as an option on the new GenArk assembly hubs. This required extending our gfServer utility to support the use of pre-computed index tables instead of the previous practice of computing those tables anew each time the server is started. On the initial alignment request from a user, a delay of 20–80 s may occur depending on whether the input sequences are DNA or protein and if there are many simultaneous requests. Once the dynamic server is primed following the first cold start, however, the new feature performs nearly as quickly as running dedicated servers full-time and consumes far less memory. As a result, we are now able to offer BLAT services on nearly all of the GenArk assemblies with only a few exceptions due to excessive genome size. Using GenArk as a template, an organization generating large batches of unique assemblies can now configure dynamic PCR and BLAT searches on their collections without requiring multiple dedicated servers for each genome.

## FUTURE PLANS

During the upcoming year, we will continue to add support for track hubs with the addition of new settings and tutorials. Another goal is to continue to develop displays that aggregate large sets of data in a digestible way, primarily with the release of features to support single-cell sequencing tracks. These tracks display as bar graphs and use the barChart format where we increased the maximum number of bars from 100 to 1000. We also plan to enhance the details page for the barChart format that displays these single-cell data with new functionality to allow easier tissue selection. New tracks will be released using these new barChart displays, working in tandem with the 70-plus datasets added the past year to our companion Cell Browser, https://cells.ucsc.edu/ (21).

## OUTREACH AND CONTACT INFORMATION

We maintain two public, moderated mailing lists for user support: genome@soe.ucsc.edu for general questions about the Genome Browser, and genome-mirror@soe.ucsc.edu for questions specific to the setup and maintenance of Genome Browser mirrors. Archives of both lists are searchable from our contacts page at https://genome.ucsc.edu/contacts.html. We can also be reached at genome-www@soe.ucsc.edu, our preferred address for questions about licenses, server error reports, or other private matters. Messages sent to that address are not archived in a publicly searchable location. We also continue to offer in-person and virtual training sessions by arrangement, https://genome.ucsc.edu/training/.

## Supplementary Material

gkab959_Supplemental_File
