# Supplementary material for: The UCSC Genome Browser database: 2022 update
Source: Nucleic Acids Res. 2021 Oct 28;50(D1):D1115–22. doi: 10.1093/nar/gkab959 (PMC8728131; doi:10.1093/nar/gkab959)
Supplement: gkab959_Supplemental_File [file gkab959_supplemental_file.pdf]

Supplementary Table 1. Annotation tracks added or updated within the last year.

KEY: N = new, U = updated, AU = automatically updated

Track Name	New / Update Status	Human Assemblies	Mouse Assemblies	Other Assemblies
DECIPHER Haploinsufficiency predictions	N	hg19		
gnomAD pext	N	hg19		
hg38 Mapping	N	hg19		
sno/miRNA	U	hg19		
TS miRNA Targets	U	hg19		
1000 Genomes Trios	N	hg38		
ClinGen Composite	N	hg38		
GENCODE V36 (primary)	N	hg38		
hg19 Mapping	N	hg38		
MANE Transcripts	U	hg38		
CADD	N	hg19, hg38		
ClinGen Research (formerly ISCA)	AU	hg19, hg38		
ClinVar Interpretations	N	hg19, hg38		
ClinVar Variants	AU	hg19, hg38		
COVID GWAS v3	N	hg19, hg38		
COVID GWAS v4	N	hg19, hg38		
dbVar Common Struct Var	N, AU	hg19, hg38		
DECIPHER CNVs and Variants	AU	hg19, hg38		
Exome Probesets	N	hg19, hg38		
GENCODE V35/V36/V37/V38 (all)	N	hg19, hg38		
Genome In a Bottle Structural Variants and Trios	N	hg19, hg38		
gnomAD (v2.1.1 update / v3.1 / v3.1.1)	N	hg19, hg38		
Locus Reference Genomic Sequences	AU	hg19, hg38		
LOVD Variants	AU	hg19, hg38		
Mastermind Variants	AU	hg19, hg38		
NCBI RefSeq	AU	hg19, hg38		

REVEL	N	hg19, hg38		
Gene Reviews	AU	hg18, hg19, hg38		
NHGRI Catalog of Published GWAS	AU	hg18, hg19, hg38		
OMIM Genes & Phenotypes	AU	hg18, hg19, hg38		
GRC Incident Database	AU	hg19, hg38	mm9, mm10	danRer10, danRer7, galGal5
Conservation (35-species on mm39)	N		mm39	
Conservation chains/nets	N		mm39	
GENCODE VM26/VM27 (all)	N		mm39	
Rhesus SNVs	N			rheMac10
Ensembl Genes v101/v104	N			47 assemblies
RefSeq Diffs	U			54 assemblies
Uniprot	AU			216 assemblies
B.1.1.7 in USA	N			SARS-CoV-2
Bloom Antibody Escape	N			SARS-CoV-2
Crowd-sourced User Annotations	N, AU			SARS-CoV-2
Human CoV	N			SARS-CoV-2
icSHAPE RNA Struct	N			SARS-CoV-2
McCoy Antibody Escape	N			SARS-CoV-2
Microdeletions	N			SARS-CoV-2
Natural Selection	N			SARS-CoV-2
Nextstrain Frequency	AU			SARS-CoV-2
Nextstrain Parsimony	AU			SARS-CoV-2
Nextstrain variants	U			SARS-CoV-2
PhyloCSF	N, U			SARS-CoV-2
Phylogeny: GISAID	U			SARS-CoV-2
Phylogeny: Public	N, AU			SARS-CoV-2
Pot. pathogenic indels	N			SARS-CoV-2
Problematic Sites	U			SARS-CoV-2
Rappuoli Serum Escape	N			SARS-CoV-2
S Protein Antibody Array	N			SARS-CoV-2
Spike Mutations	N			SARS-CoV-2
Swift Primers	N			SARS-CoV-2
Uniprot	AU			SARS-CoV-2

Vaccines track	N			SARS-CoV-2
Variants of Concern	N			SARS-CoV-2
Weizman ORFs	N			SARS-CoV-2
Whelan 21 Antibody Escape	N			SARS-CoV-2
